# Effect of Different Movement Speed Modes on Human Action Observation: An EEG Study

**DOI:** 10.3389/fnins.2018.00219

**Published:** 2018-04-05

**Authors:** Tian-jian Luo, Jitu Lv, Fei Chao, Changle Zhou

**Affiliations:** Fujian Provincal Key Lab of Brain-Inspired Computing, Department of Cognitive Science, School of Informatics, Xiamen University, Xiamen, China

**Keywords:** action observation, different speed modes, hMNS activations, ERD suppressions, BCI

## Abstract

Action observation (AO) generates event-related desynchronization (ERD) suppressions in the human brain by activating partial regions of the human mirror neuron system (hMNS). The activation of the hMNS response to AO remains controversial for several reasons. Therefore, this study investigated the activation of the hMNS response to a speed factor of AO by controlling the movement speed modes of a humanoid robot's arm movements. Since hMNS activation is reflected by ERD suppressions, electroencephalography (EEG) with BCI analysis methods for ERD suppressions were used as the recording and analysis modalities. Six healthy individuals were asked to participate in experiments comprising five different conditions. Four incremental-speed AO tasks and a motor imagery (MI) task involving imaging of the same movement were presented to the individuals. Occipital and sensorimotor regions were selected for BCI analyses. The experimental results showed that hMNS activation was higher in the occipital region but more robust in the sensorimotor region. Since the attended information impacts the activations of the hMNS during AO, the pattern of hMNS activations first rises and subsequently falls to a stable level during incremental-speed modes of AO. The discipline curves suggested that a moderate speed within a decent inter-stimulus interval (ISI) range produced the highest hMNS activations. Since a brain computer/machine interface (BCI) builds a path-way between human and computer/mahcine, the discipline curves will help to construct BCIs made by patterns of action observation (AO-BCI). Furthermore, a new method for constructing non-invasive brain machine brain interfaces (BMBIs) with moderate AO-BCI and motor imagery BCI (MI-BCI) was inspired by this paper.

## 1. Introduction

Human mirror neuron system (hMNS) components activate the sensorimotor, parietal, and occipital cortices when a human performs, observes, or imitates an action (Rizzolatti, 2005; Tanji et al., 2015). Initial studies of the hMNS focused on motor imitation and learning, as well as action understanding and observation (Rizzolatti and Sinigaglia, 2010; Pineda et al., 2013). Furthermore, the hMNS has gradually come to be regarded as crucial for social skills such as understanding the intensions and emotional states of others (Blakemore and Decety, 2001; Schulte-Rüther et al., 2007). Action observation (AO) was considered one of the primary ways of inducing hMNS activations in early studies (Caspers et al., 2010; Mukamel et al., 2010; Rozzi and Fogassi, 2017). The modulation of hMNS recruitments by AO has positive effects on cognitive psychology and sport rehabilitation for creating scientific recovery strategies (Franceschini et al., 2012). hMNS activations by AO also provide another way to construct a brain computer/machine interface (BCI) for mechanical control and medical auxiliary purposes (Neuper et al., 2009). A BCI constructs an alternative method for communicating between human and computers/machines. In BCIs, the motor related BCI was constructed by the variation patterns in mu (8–12 Hz) and beta (18–25 Hz) bands power extracted by electroencephalography (EEG) when a human image, observer, or execute limb/leg movements. Variations in band power in the two rhythms are referred to as event-related desynchronization (ERD) suppression. Hence, the ERD suppression will be caused by motor imagery (MI) and AO. In fact, the definitions of AO tasks influence the degree of ERD suppression. However, the relationships between action definitions for AO and hMNS activations are still unknown from a scientific view.

In general, in the movements designing of an AO therapy for stroke patients or a BCI system, the parameters and affecting factors will influence the performance of therapy or BCI accuracy. In fact, the affecting factors will be controlled if we explore the disciplines of the relationships between movements for AO and the hMNS activations. Hence, the the research of the affecting factors of movements for action observation will do favor for designing a AO therapy scheme or an AO-BCI system. Most prior studies offer different hypotheses regarding the relationships between movements for AO and the hMNS activations (Iacoboni et al., 2005; Filimon et al., 2007; Newman-Norlund et al., 2007). Since many AO studies are based in cognitive psychology and sports psychology, while few are based in rehabilitation and BCI research, the distribution and degree of hMNS activations remain controversial with respect to several features of AO (Perry et al., 2010; Vogt et al., 2013). The mode of AO movements is the main factor that limits hMNS and motor system activation. Several neuro-physiological studies indicate that ongoing movement plays an important role in triggering hMNS responses when activations are induced by AO (Maranesi et al., 2013). Aside from the condition of ongoing movements, other studies hypothesize that the manifestations of actions in AO tasks influence hMNS activations. In a study of action meaning (Agnew et al., 2012), compared with meaningless movements, meaningful movements caused higher hMNS activations. For example, lipreading of speech is one of the meaningful movements. Researches on lipreading showed lipreading will enhance speech perception and speech recognition (Summerfield, 1992; Silsbee and Bovik, 1996). Lipreading enhancement in young deaf children will help auditory speech perception (Geers, 1994). Lipreading and audiovisual speech integration are used in therapy of autism (Smith and Bennetto, 2007). Hence, the researches on action meaning will be useful for the therapy, rehabilitation, and neuroscience. In addition, the degree of movement complexity in an mode of AO movement has also been found to affecting hMNS activations (Biagi et al., 2010; Gatti et al., 2017). Moreover, additional variables that modulate hMNS activation and ERD suppression, such as experience and familiarity, have also been studied (Calvo-Merino et al., 2006; Bello et al., 2014).

Although the factors affecting the relationships between movements for AO and hMNS activation have been explored by the above studies, hMNS specificity and its full significance remain uncertain. The definitions of AO tasks clearly influence the degree of ERD suppression (Orgs et al., 2008). Furthermore, the manifestations of AO tasks and their stimuli affect attentional resources and hMNS activation (Oberman et al., 2007). Since the majority of affecting factors are related to the modes of AO movements, explorations of the relationships between movements for AO and hMNS activation are translated into the relationships between movement modes and the degree of ERD suppressions. Speed, complexity, magnitude, and individual proficiency are well-characterized factors known to affect movement modes, and have been explored in previous studies (Abdi and Williams, 2010; Biagi et al., 2010; Agnew et al., 2012; Maranesi et al., 2013; Bello et al., 2014). However, to the best of our knowledge, the present study is the first to investigate the effect of speed during AO. If the speed affecting factor is well-explored for AO among individuals, the designing for therapy scheme and AO-BCI will be more accurate for individuals. In addition, since the parameters and affecting factors will be well controlled in designing AO-BCI, but not in designing MI-BCI, the performance of AO-BCI will be better than MI-BCI. Furthermore, based on BCI system, the brain computer/machine brain interfaces (BMBIs) are capable of bidirectional communication with the brain. The interfaces consist of efferent and afferent modules. The efferent modules decode motor intentions by the variation patterns in mu and beta bands power, like conventional BCIs. The afferent modules encode feedback about the interactions of the machine through patterns of intracortical microstimulation. In general, the efferent modules are always designed by patterns of motor imagery. Meanwhile, the afferent modules can't be designed by patterns of MI. However, the afferent modules will be designed by patterns of action observation. When the efferent modules by MI drive a machine performing movements, the feedback by observation to activate hMNS will cause ERD suppression to design afferent modules. Today's BMBIs are constructed without considering the affecting factors of movements, so the performance is bad. However, if the speed and other affecting factors are considered for designing BMBIs, the performance will be better.

EEG (Lei et al., 2015; Abbott, 2016) has been widely used to construct BCI systems for exploring hMNS activations due to its high temporal resolution. In our study, the experimental movements were made by a humanoid robot at four different speed modes for AO tasks. A humanoid robot platform provides identical appearance, background and complexity to eliminate other effects. All four speed modes, slow (0.25 Hz), moderate (1.25 Hz), fast (2.75 Hz), and finalistic (4 Hz), were defined by questionnaires administered to 26 participants. Another six subjects were asked to attend AO experiments and an MI experiment for comparison. All eight stimuli (left movement and right movement at four speed modes) were randomly presented to all subjects in AO experiments, and another two stimuli were randomly presented to the same subjects in MI experiments. Both experiments are designed by a BCI form to validate the relationships between speed mode of AO and the hMNS activations. Due to the high temporal resolution, quantitative EEG (Lei et al., 2015; Abbott, 2016) is used as a convenient and cheap method to explore hMNS activations by measuring ERD suppressions (Oberman et al., 2007; Orgs et al., 2008). The ERD suppressions in EEG data are analyzed using a series of BCI methods and spatio-spectral-temporal characteristics. The following three main points are analyzed and discussed in this study:

1. The brain regions involved in AO, such as the sensorimotor and occipital regions, are compared since studies on activation regions for AO have paradoxical conclusions (Perry et al., 2010; Frenkel-Toledo et al., 2013).
2. The patterns of the relationships between AO speed modes and hMNS activations. If the patterns are clearly explored by experiments, individual rehabilitation strategies with specific optimal speeds will be precisely designed for individuals.
3. Application to brain machine brain interface (BMBI). The average pattern of all individuals will show optimal speed ranges that are best suited to constructing AO-BCIs. Moreover, a well-established non-invasive AO-BCI with a high rate of accuracy and information transfer rate (ITR) will help to build BMBIs.

## 2. Materials and methods

### 2.1. Ethics statement

This study was carried out in accordance with the recommendations of the guideline of EEG experiment, Xiamen University ethics committee with written informed consent from all subjects. All subjects gave written informed consent in accordance with the Declaration of Helsinki. The protocol was approved by the Xiamen University ethics committee.

### 2.2. Participants

Six right-handed healthy human subjects (five male and one female, mean age: 24.6 years, *SD* = 1.46) were invited to participate in AO and MI experiments with payment. All subjects had no history of neurological disease and either normal or corrected-to-normal vision. Subjects also did not have any professional experience with AO or MI experiments. In accordance with the principles of the Helsinki Declaration, all subjects provided written informed consent approved by the Xiamen University.

### 2.3. Stimuli design

**Figure 1:**
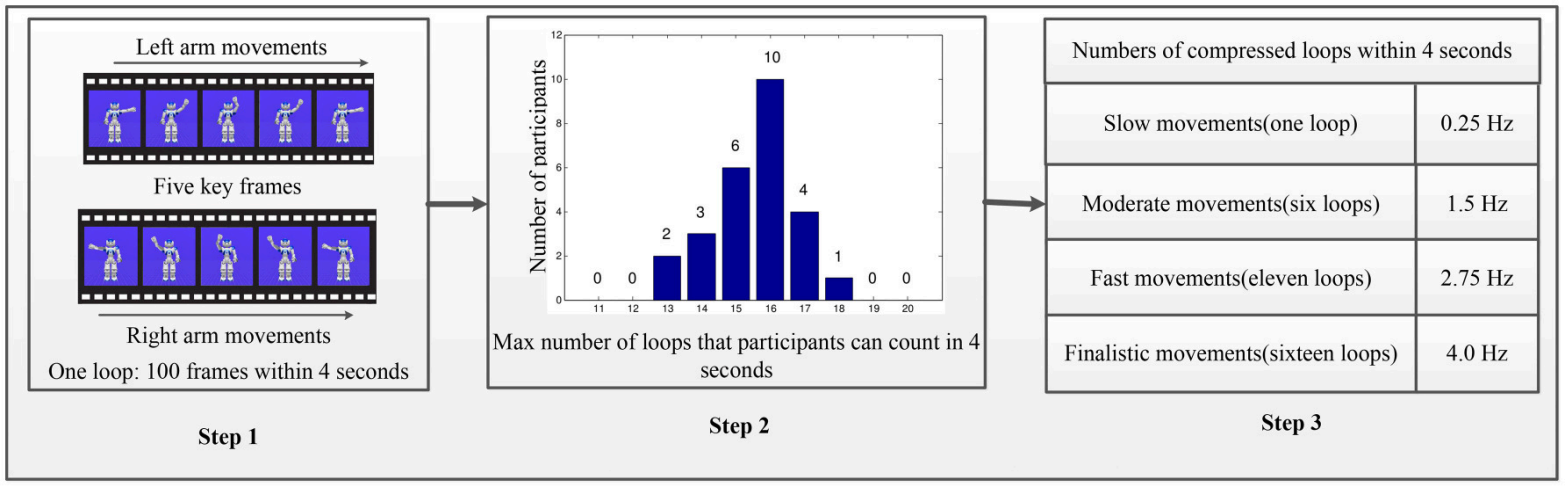
Stimuli design of AO in different speed modes. The Step 1 contains one loop of periodic-swinging arm movement includes 100 frames over four seconds because the platform has a fixed frame rate of 25 fps. The Step 2 summarizes the results of the correct maximum loop number counted by each participant. The Step 3 defines the role of speed modes in the pattern of relationships between AO tasks and hMNS activations. As well as for simplicity and convenience, four different loops with a constant interval were constructed. Step 1: One loop for AO at standard speed. Step 2: Twenty-six participants count loops at higher speed modes to determine the max (16 loops). Step 3: Select four speed modes for experiments by equi-partition.

As shown in Figure 1, stimuli for AO in different speed modes were performed by a humanoid robot. AO stimuli were presented to all subjects in 12 sessions as experiment 1. Afterwards, an MI comparison experiment in which two guidance arrows were presented to the same subjects in three sessions was performed as experiment 2.

#### 2.3.1. Experiment 1: AO experiments in different speed modes

In previous studies, AO experimental paradigms were designed with sequential video clips of human arm, hand, or finger movements presented to subjects (Frenkel-Toledo et al., 2014; Gatti et al., 2017). However, our experiments were designed to explore speed factor during AO. Because human movements feature unconstant speed, angle, and magnitude across repetitive experiments, the inconsistency of the variables will introduce confounding factors that disturb the analyses of hMNS activations. To eliminate such confounding factors, a fine-tuning parameter-setting paradigm must be incorporated into the design. Based on the anthropomorphic and relevant AO studies (Press, 2011), the hMNS is strongly activated by observations of both human and non-human agents (e.g., humanoid robots). Human or non-human agents do not differ significantly in their ability to activate the hMNS in AO studies involving humans and humanoid robots. Since the robot platform features an operating system for controlling all movement parameters, a humanoid robot platform was used in the design of our AO experimental paradigms.

Based on the “NAO Choregraphe 1.14 platform” (Pot et al., 2009), periodic-swinging movements of the robot's left/right arm were utilized as AO tasks in experiment 1. As shown in Figure 1 (Step 1), one loop of periodic-swinging arm movement includes 100 frames over 4 s because the platform has a fixed frame rate of 25 fps. Five key frames for both the left and right arms show the same procedure of the periodic movements. The robot stretches its arm at the beginning of the loop and waves its arm up to the head during half of the loop. The remainder of the loop consists of the same procedure in the opposite direction. The platform ensures that the robot's left/right-arm movements maintain a constant speed, angle, and magnitude. The entity body of the robot was presented to subjects in order to distinguish left-arm or right-arm movements during AO using EEG-based BCI analysis methods (Bauer and Gharabaghi, 2015). The robot's arms are dynamic, while the other parts of the robot remain static to keep the speed variable clear and unique.

Theoretically, more speed modes will be better for the results. However, due to the limitation of participants and the experimental platform, we only explore the rough pattern of the factor affections for simplicity and convenience. Four different loops with a constant interval were constructed as shown in Figure 1 (Step 3). For simplicity, the four incremental speed movements are defined as “slow movement,” “moderate movement,” “fast movement,” and “finalistic movement.” In Figure 1 (Step 1), one loop of 100 frames over 4 s is defined as “slow movement.” Due to time duration in AO experiments (4 s per trial), we obtain higher movement speed modes by adding number of loops, and compressing multi-loops within 4 s. To identify a robust and suitable number of multi-loops for “finalistic movement,” 26 participants were invited to watch 10 different looped (11–20) video clips that were compressed into 4 s. Participants were asked to count the number of loop in each video clip. The results from all participants were then compared with ground truth to identify the maximum loop number for each participant. Figure 1 (Step 2) summarizes the results of the correct maximum loop number counted by each participant. The results show that 16 loops was the maximum loop number for all of the participants. Therefore, “finalistic movement” was defined as 16 loops compressed into 4 s. “Moderate movement” was set to six loops, and “fast movement” was set to 11 loops, by the principle of equipartition. In all, experiment 1 gives four different speed modes of AO to explore the role of speed. All four speed modes were presented to subjects during AO in experiment 1.

#### 2.3.2. Experiment 2: relevant MI experiment

After experiment 1, all six healthy human subjects were asked to attend experiment 2. The MI experiment was compared with the AO experiment. Following the design criteria of MI experimental paradigms (Tangermann et al., 2012), two guidance arrows were defined in experiment 2 to guide the imagined movement direction. In experiment 2, the subjects were asked to imagine his/her-self as performing the same movement as the humanoid robot in experiment 1. Both MI guidance arrows used in experiment 2 were presented to all subjects after experiment 1.

### 2.4. Experimental procedures

**Figure 2:**
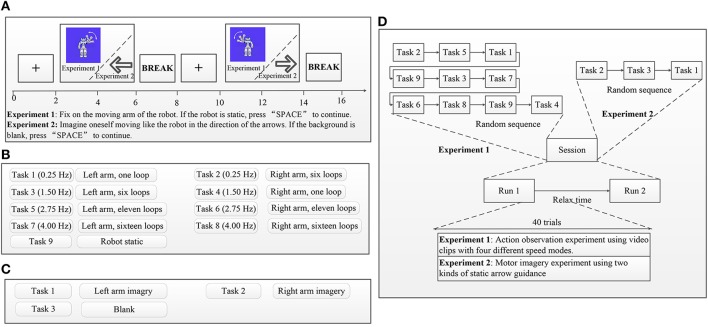
Experimental procedures. In each trial, initial instructions guided subjects to simply observe the robot's movements (experiment 1) or guidance arrows (experiment 2) on the screen and a manually respond as rapidly as possible whenever the catch trial static robot (experiment 1) or blank (experiment 2) is presented. Each trial started with a fixation cross presented in the middle of a gray screen. The duration of the fixation cross was 2,000 ms. After presentation of the fixation cross, stimuli were presented in the middle of the gray screen for 4,000 ms to investigate participants' ERD suppressions via EEG recordings and were followed by a 2,000-ms pause as a break. Each of the eight AO stimuli (left and right in four speed modes) was randomly presented four times, and the catch trial stimulus was randomly presented eight times within one session in Experiment 1. Each of the two MI stimuli (left and right) was randomly presented four times, and the catch trial stimulus was randomly presented eight times within one session in Experiment 2. **(A)** Example trial sequence for experiment 1 and experiment 2. **(B)** Task descriptions for experiment 1. **(C)** Task descriptions for experiment 2. **(D)** Example session sequence for experiment 1 and experiment 2.

As shown in Figure 2, the experimental procedures for AO in different speed modes and MI were divided into trials and sessions. EEG recordings were set for both AO and MI experiments.

#### 2.4.1. Trial procedures

Influenced by conventional AO/MI experimental trial setups (Calvo-Merino et al., 2004; Wang et al., 2012), the presentation of a trial is shown in Figure 2A. In each trial, initial instructions guided subjects to simply observe the robot's movements (experiment 1) or guidance arrows (experiment 2) on the screen and a manually respond as rapidly as possible whenever the catch trial static robot (experiment 1) or blank (experiment 2) is presented. Subjects were also asked to minimize eye movements and keep their eyes fixed on the monitor when observing the stimuli. Manual responses to the catch trial were input by pressing the “SPACE” button. Each trial started with a fixation cross presented in the middle of a gray screen. The duration of the fixation cross was 2,000 ms. After presentation of the fixation cross, stimuli were presented in the middle of the gray screen for 4,000 ms to investigate participants' ERD suppressions via EEG recordings and were followed by a 2,000-ms pause as a break. If the presentation was an AO stimulus (experiment 1), subjects were asked to focus on the moving arm of the robot; otherwise, when the presentation was an MI stimulus (experiment 2), subjects were asked to imagine him/her-self moving like the robot by following the guidance arrows. Subsequent trials followed the same procedure for both experiment 1 and experiment 2.

#### 2.4.2. Session procedures

In experiment 1, each subject completed 12 AO sessions within 6 days, whereas in experiment 2, each subject completed three MI sessions within 2 days. Data recordings for one session occurred in the morning, with another session taking place later in the afternoon the same day. As shown in Figure 2D, each session comprised two tasks, and each task was divided into 40 trials. A relaxation time to prevent fatigue was determined by subjects between the two tasks. Experiment 1 comprised eight AO stimuli and one catch trial stimulus, which are described in Figure 2B. Each of the eight AO stimuli (left and right in four speed modes) was randomly presented four times, and the catch trial stimulus was randomly presented eight times within one session (See Figure 2D). Experiment 2 comprised two MI stimulus arrows and one blank catch trial stimulus, which are described in Figure 2C. Each of the two MI stimuli (left and right) was randomly presented four times, and the catch trial stimulus was randomly presented eight times within one session (See Figure 2D). Response times to the catch trial stimulus were recorded for both experiments 1 and 2 because the response times help confirm attentional states during AO and MI experiments.

#### 2.4.3. EEG recordings

Each subject was seated individually in a comfortable arm chair in front of a computer monitor in a dimly lit, sound-attenuated room and asked to read the instructions carefully on the screen. AO stimuli (experiment 1) and MI stimuli (experiment 2) were displayed on a DELL P2314H LCD monitor with a refresh rate of 60 frames/s. Displays were controlled by the psychology software tool “E-Prime 2.0.” The distances between monitor and the subjects were set at 100 cm by following the BCI experimental criterion to prevent other confounding factors in the hMNS activation (Onishi et al., 2017). During the entire run of both experiments, a spatially homogeneous gray background with a luminance of 38 cd/m2 was enabled. EEG recordings for all subjects were performed on a “NeuroScan SynAmps2” device with a “Neuroscan QuikCap international 10–20 system.” EEG signals were referenced to the nose, grounded at the frontal position (Fpz), and sampled at 250 Hz. After data acquisition, the preprocessing operations on the signals for notch filtering and bandpass filtering were 48–52 and 0.1–100 Hz, respectively. All 64 electrode impedances in QuikCap were kept below 5kω during the experiment. Moreover, the horizontal and vertical EOG signals were recorded. The EOG signals were used to correct for the influence of blinking and eyeball movements.

### 2.5. Analysis methods

**Figure 3:**
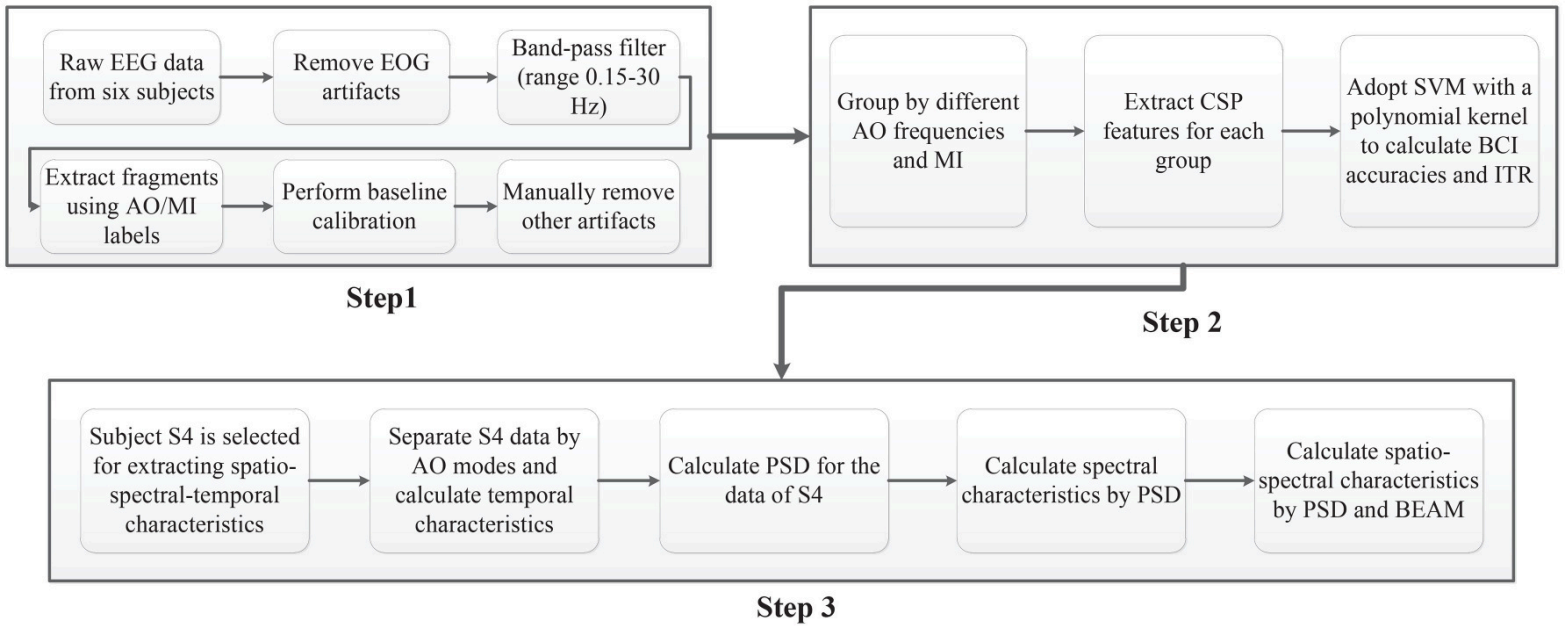
EEG data analysis procedures. Step 1: Pre-processing. Step 2: BCI analyses. Step 3: Extracting spatio-spectral-temporal characteristics.

As shown in Figure 3, three main EEG data analyses procedures were utilized in this study. Figure 3 (Step 1) illustrates the preprocessing procedure. Raw EEGs from six subjects were first corrected for EOG artifacts via blind component separation. Then, a band filter ranging from 0.15 to 30 Hz was adopted for EEG data, since the effective parts are above 0.15 Hz and under 30 Hz. By using AO (experiment 1) and MI (experiment 2) labels recorded in “NeuroScan,” fragments of each experimental condition were extracted for all subjects. After fragment extraction, a baseline calibration method to prevent deviation and a manual removal method to reducing artifacts were performed on all fragments.

Recent studies first found that hMNS activations initially occurs in the sensorimotor region of the brain during AO (Perry et al., 2010). The ERD suppressions in the sensorimotor region then extend to attentional resources, which cause more intense hMNS activations in the occipital region (Frenkel-Toledo et al., 2013). Likewise, ERD suppressions caused by MI are first found in the sensorimotor region and then extend to global cerebral activity. To explore the affected regions of the brain during AO, the patterns of relationships between AO speed modes and hMNS activations, as well as to compare AO and MI to construct a BMBI, the sensorimotor, occipital and “sensorimotor + occipital” regions were adopted for BCI analyses of both AO and MI experiments. Potentials covering central-parietal regions C3, C1, Cz, C2, C4, P3, P1, Pz, P2, and P4 were chosen for the sensorimotor region, while potentials covering parietal-occipital regions PO5, PO3, POz, PO4, PO6, O1, Oz, and O2 were chosen for the occipital region. The “sensorimotor + occipital” region included both sets of potentials.

As illustrated in Figure 3 (Step 2), the procedure for BCI analyses comprised four steps. First, left fragments and right fragments were grouped by identical AO speed (experiment 1). Second, all groups of fragments were presented to extract filter bank common spatial pattern (FB-CSP) features (Ang et al., 2012). Since ERD suppressions are modulated by the power variations of the left and right cerebral hemispheres, an FB-CSP algorithm was adopted to extract power variations using a bank of filters and an optimization method. The optimal CSP eigen-vectors were set to *S* = 4 based on experience. Third, the principal component analysis (PCA) algorithm of a linear dimensionality reduction technique was adopted to reduce feature dimensions (Abdi and Williams, 2010). Because of the high sampling rate in EEG recordings, FB-CSP features have a high number of dimensions that are hard to classify by machine-learning models. The PCA algorithm looks for the highest variability in FB-CSP features by projecting original features to lower dimensions. The optimal PCA was set to *M* = 10 for the regulation of “99%” of the variability. Finally, the dimension-reduced FB-CSP features were incorporated into the support vector machine (SVM) classifier for BCI classification (Sardouie and Shamsollahi, 2012). The SVM classifier is a common BCI classifier with excellent generalizability by searching for the largest margin of a decision hyperplane that will strictly classify the unseen test data. The reductive features were incorporated into SVM classifiers with a polynomial kernel. A 6^*^6 cross-validation strategy was applied to train the SVM classifiers for all five groups. Since each group has 392 trials of effective EEG fragments with a random order for left and right, 320 trials were provided as training data, and the remaining 64 trials as evaluation data in one loop of evaluation.

Figure 3 (Step 3) displays the procedure for extracting spatio-spectral-temporal characteristics. Subject 4, who had the best BCI accuracies, was chosen for the analyses of these characteristics. The temporal characteristics were first extracted by calculating mean voltages of EEG fragments among four different speed AO modes (both for left and right). Then, a power spectral density (PSD) (Demandt et al., 2012) algorithm with parameters of specific rhythm ranges was used for each average fragment to obtain spectral characteristics. The spectral characteristics were drawn by the “eeglab 14.1.1” tool as “ERD images” (Delorme and Makeig, 2004). Brain electrical activity maps (BEAMs) (Duffy et al., 1979) were imported to obtain the positions of the potentials, and the spatio-spectral characteristics were drawn by the “eeglab 14.1.1” tool.

In addition, the response times of catch trial stimuli as feedback behavioral data were analyzed by statistical analyses. Mean time values for all 15 sessions (Sessions 1–12 for experiment 1 and sessions 13–15 for experiment 2) were calculated for all subjects to obtain the attentional conditions of all sessions by calculating mean values and standard deviations among all sessions.

## 3. Results

### 3.1. Feedback behavioral data

Catch trial stimuli appear 16 times in each session, and we have recorded the response times for all catch trial stimuli in each session. Tables 1, 2 illustrate the mean response times within one session for experiment 1 and experiment 2, respectively. The mean time and standard deviations among all sessions in both experiments are also presented.

**Table 1:** Statistical analyses of response times for Experiment 1 (ms).

**Sessions**	**Subject 1**	**Subject 2**	**Subject 3**	**Subject 4**	**Subject 5**	**Subject 6**
Session 1	1760.53	868.97	868.97	1521.47	1716.25	1007.38
Session 2	1087.91	912.84	696.41	1411.47	1254.91	1706.56
Session 3	1201.75	993.75	621.06	1031.94	1695.63	1436.28
Session 4	1141.13	890.38	642.44	1102.09	1783.66	1093.78
Session 5	1243.38	943.69	518.03	1052.81	1644.91	1306.13
Session 6	1175.63	1047.78	749.00	1625.84	1701.25	1577.13
Session 7	1115.03	853.84	829.81	1306.88	1304.25	1420.00
Session 8	1417.50	818.72	777.56	1367.94	1562.38	1513.63
Session 9	1417.50	699.81	763.16	1655.03	1301.03	1268.88
Session 10	1392.31	660.88	759.81	1509.13	1284.75	1204.16
Session 11	1339.03	557.09	828.41	1509.13	1450.06	1181.03
Session 12	1582.00	656.63	790.31	1348.56	1526.16	1181.09
Average	1310.30	825.37	727.84	1370.19	1518.77	1324.67
Standard	201.81	150.67	92.55	213.45	194.04	208.90

*The average accuracies and standard deviations of response times were calculated for experiment 1*.

**Table 2:** Statistical analyses of response times for Experiment 2 (ms).

**Sessions**	**Subject 1**	**Subject 2**	**Subject 3**	**Subject 4**	**Subject 5**	**Subject 6**
Session 13	234.44	238.09	183.81	298.13	286.75	193.63
Session 14	284.31	282.72	219.16	307.94	317.06	221.09
Session 15	258.03	262.84	204.41	287.88	262.31	237.88
Average	258.93	261.22	202.46	297.98	288.71	217.53
Standard	24.95	22.36	17.76	10.03	27.43	22.34

*The average accuracies and standard deviations of response times were calculated for experiment 2*.

As shown in Table 1, the average response times among all sessions of experiment 1 were within 2,000 ms, and the corresponding standard deviations were within 100–200 ms. These results demonstrate that all subjects exhibited focused attention during AO experiments and EEG recordings. Therefore, EEG recordings in experiment 1 were free from attention artifacts. The standard deviations of all subjects were within a similar range, indicating that the responses to stimuli were stable. The response times of Subjects 2 and 3 had lower averages and standard deviations, which may be related to their younger age, as this tends to be related to faster response times. Since our behavioral data conform to experimental principles, the EEG data of experiment 1 were considered objective and appropriate for analyses. Similarly, as illustrated in Table 2, the average response time among all sessions of experiment 2 were within 300 ms, and the corresponding standard deviations were within 10 and 20 ms. These results conform with the response times of MI experiments, indicating that the EEG recordings in experiment 2 were objective and appropriate for analyses. Compared with Tables 1, 2, the blank catch trial stimuli was easier to recognize than a static robot, and the individual differences in the response times of younger subjects has less influence on the MI experiments.

### 3.2. BCI analyses of AO and MI experiments

**Figure 4:**
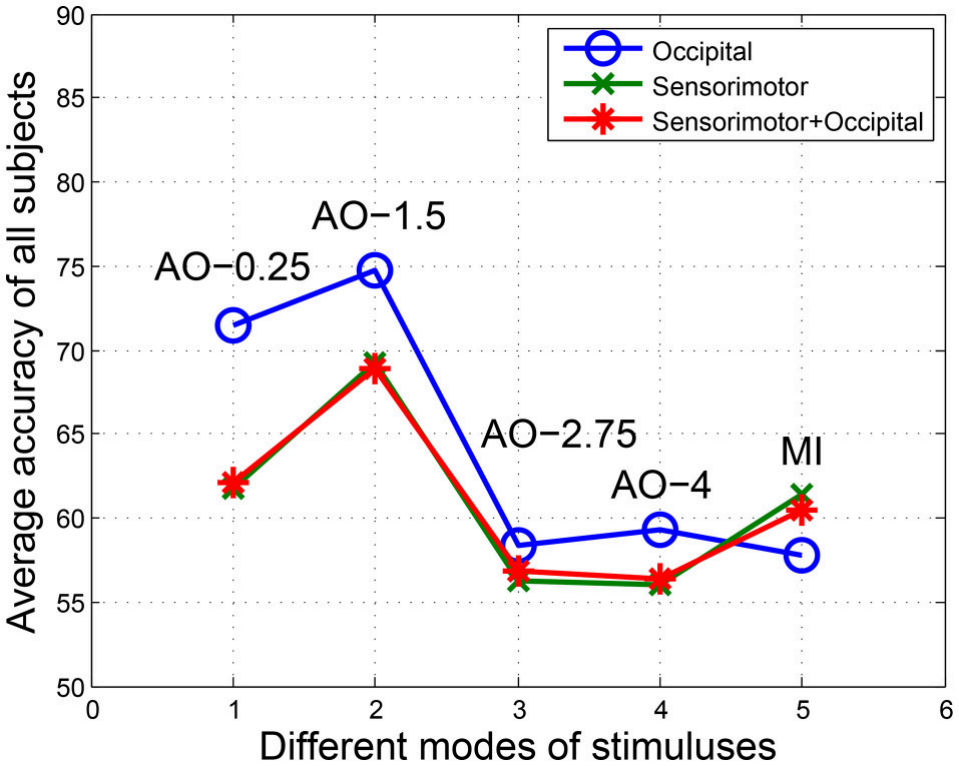
The average accuracies of all modes in all regions. Potentials covering central-parietal regions C3, C1, Cz, C2, C4, P3, P1, Pz, P2, and P4 were chosen for the sensorimotor region, while potentials covering parietal-occipital regions PO5, PO3, POz, PO4, PO6, O1, Oz, and O2 were chosen for the occipital region. The “sensorimotor + occipital” region included both sets of potentials.

After preprocessing, the EEG fragments (left and right) of four speed AO modes and MI modes were used for BCI analyses. In FB-CSP feature extraction, the bank of bank filters following a previous study are shown in Table 3. Table 3 illustrates the BCI analysis results for three regions in four AO speed modes and MI mode. The average accuracies and standard deviations of BCI classification and ITR were calculated. To display the comparison of the results, Figure 4 shows the average accuracies of all modes in all regions.

**Table 3:** The subbands of band-pass filters in FB-CSP feature extraction.

**Subbands**	***fb*_1_**	***fb*_2_**	***fb*_3_**	***fb*_4_**	***fb*_5_**	***fb*_6_**	***fb*_7_**	***fb*_8_**	***fb*_9_**	***fb*_10_**
Frequency(Hz)	[0, 4]	[4, 8]	[8, 12]	[12, 16]	[16, 20]	[20, 24]	[24, 28]	[28, 32]	[32, 36]	[36, 40]

*A bank of 10 band-pass filters from 0 to 40 Hz without overlaps are set*.

From the BCI analysis results in Table 4 and Figure 4, we see four findings:

1. For the accuracies in the occipital region, the results were AO-0.25 Hz (71.44%), AO-1.5 Hz (74.78%), AO-2.75 Hz (58.42%), and AO-4 Hz (59.29%). However, the results were AO-0.25 Hz (61.76%), AO-1.5 Hz (69.27%), AO-2.75 Hz (56.29%), and AO-4 Hz (55.99%) in the sensorimotor region. Similarity, the accuracies of the “sensorimotor+occipital” were AO-0.25 Hz (62.07%), AO-1.5 Hz (68.92%), AO-2.75 Hz (56.81%), and AO-4 Hz (56.33%). Considering the results across all regions, the BCI accuracies from increasing speed modes of AO presented a pattern of initially increasing and then decreasing to a stable level.
2. Comparing AO-BCI accuracies between occipital and sensorimotor regions, the results were 71.44 and 61.76% at AO-0.25 Hz, 74.78 and 69.27% at AO-1.5 Hz, 58.42 and 56.29% at AO-2.75 Hz, and 59.29 and 55.99% at AO-4 Hz. AO in any speed mode had a more significant BCI accuracy in the occipital region than in the sensorimotor region. However, the accuracies of MI-BCI were 57.72% in the occipital region and 61.35% in the sensorimotor region. MI mode had a more significant BCI accuracy in the sensorimotor region than in the occipital region.
3. The BCI accuracies in the “sensorimotor+occipital” region were at a similar level to those in the sensorimotor region. The results were 62.07 and 61.76% at AO-0.25 Hz, 68.92 and 69.27% at AO-1.5 Hz, 56.81 and 56.29% at AO-2.75 Hz, and 56.33 and 55.99% at AO-4 Hz. However, a large difference was detected between the “sensorimotor+occipital” and occipital regions. The results were 62.07 and 71.44% at AO-0.25 Hz, 68.92 and 74.78% at AO-1.5 Hz, 56.81 and 58.42% at AO-2.75 Hz, and 56.33 and 55.99% at AO-4 Hz. These results suggested the BCI accuracy was more robust in the sensorimotor region and was resistant the influence of other regions. Moreover, the higher BCI accuracies in the occipital region may be influenced by factors other than hMNS activations.
4. Because the standard deviations of all experimental modes in all regions were within the range of [0, 10], AO in different speed modes and MI mode have similar robustness. The average standard deviations in all regions between AO modes showed that AO-0.25 Hz (5.69) and AO-1.5 Hz (5.80) have lower robustness than AO-2.75 Hz (2.98) and AO-4 Hz (2.13). Since ITR presents the efficiency of BCI, the average results of ITR in all regions showed that AO-1.5 Hz (2.01) had a significantly higher efficiency than AO-0.25 Hz (1.10), AO-2.75 Hz (0.23), AO-4 Hz (0.24), and MI (0.43). AO in moderate-speed mode was more suitable for the design of BCIs.
5. The average accuracies have the same pattern of initially increasing and then decreasing to a stable level. In the results between subjects, subjects “S1, S3, S4” correspond to the average pattern, but subjects “S2 and S5” not correspond to the average pattern from AO-0.25 Hz to AO-4 Hz. However, subjects “S2 and S5” have approximate accuracies in AO-0.25 Hz and AO-4 Hz. This because different individuals have different patterns of speed modes, and our study was researched on rough speeds due to limitations of device and platform.

**Table 4:** The BCI analysis results for three regions for four AO modes and MI modes.

**Subjects**	**AO-0.25 Hz**	**AO-1.5 Hz**	**AO-2.75 Hz**	**AO-4.0 Hz**	**MI**
**OCCIPITAL REGION**					
S1	69.27	79.17	54.43	59.38	60.16
S2	77.08	69.79	59.11	59.38	59.90
S3	69.01	76.30	54.95	62.24	57.03
S4	69.27	81.77	68.75	61.46	57.29
S5	67.71	65.36	56.25	54.43	58.85
S6	76.30	76.30	57.03	58.85	53.13
Average	71.44	74.78	58.42	59.29	57.72
Standard	4.12	6.10	5.33	2.73	2.60
ITR (bits/min)	2.06	2.78	0.31	0.38	0.26
**SENSORIMOTOR REGION**					
S1	69.01	75.52	56.77	56.77	55.47
S2	72.14	68.49	57.03	57.29	57.03
S3	56.77	67.45	58.33	55.47	54.69
S4	54.69	67.19	56.51	52.60	54.69
S5	60.42	70.83	55.21	56.77	67.97
S6	57.55	66.15	53.91	57.03	78.29
Average	61.76	69.27	56.29	55.99	61.35
Standard	7.14	3.45	1.54	1.77	9.72
ITR (bits/min)	0.61	1.65	0.17	0.16	0.56
**“OCCIPITALx + SENSORIMOTOR” REGION**					
S1	55.21	73.96	56.25	56.25	55.47
S2	71.61	69.53	60.68	58.85	55.21
S3	65.10	59.11	54.43	58.33	56.25
S4	58.33	81.25	56.77	54.17	55.47
S5	62.76	64.32	56.77	55.47	61.72
S6	59.38	65.36	55.99	54.95	78.39
Average 62.07	68.92	56.81	56.33	60.42
Standard	5.82	7.84	2.08	1.88	9.14
ITR (bits/min)	0.64	1.59	0.20	0.17	0.47

*Potentials covering central-parietal regions C3, C1, Cz, C2, C4, P3, P1, Pz, P2, and P4 were chosen for the sensorimotor region, while potentials covering parietal-occipital regions PO5, PO3, POz, PO4, PO6, O1, Oz, and O2 were chosen for the occipital region. The “sensorimotor + occipital” region included both sets of potentials. The average accuracies and standard deviations of BCI classification and ITR were calculated*.

These five findings will help us understand affected regions in the brain during AO and the patterns of relationships between AO speed modes and hMNS activations. By selecting the optimal speed mode of AO, we also solved the problem of BMBI. These findings and conclusions are discussed below.

### 3.3. Spatio-spectral-temporal characteristics of AO experiments

To further analyse the detailed characteristics, the best-performing data, S4, was chosen to extract the spatio-spectral-temporal characteristics of AO modes.

#### 3.3.1. Temporal characteristics

**Figure 5:**
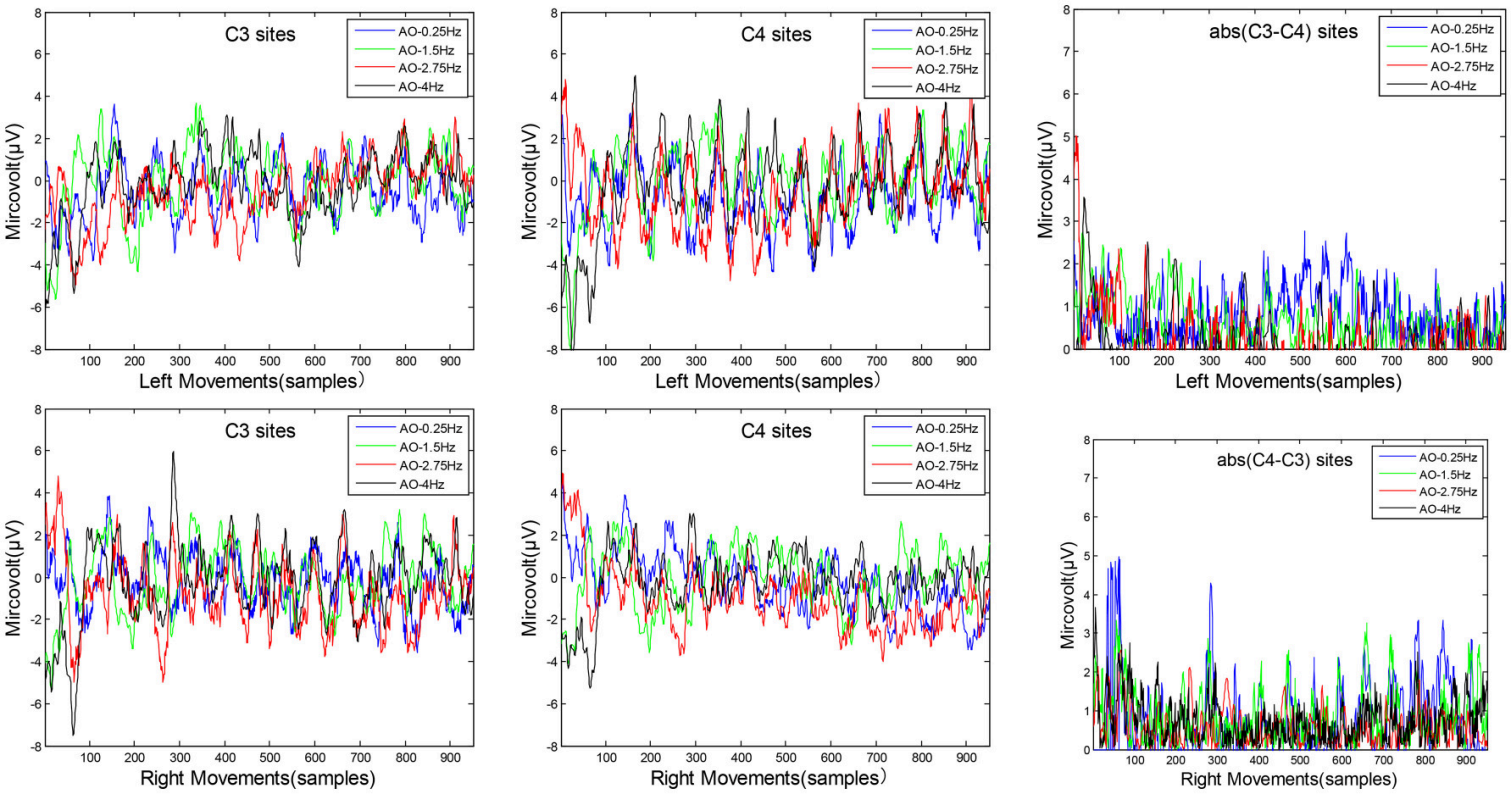
The temporal characteristics of AO in four different speed modes. Two key sites, C3 and C4 from the sensorimotor region, were chosen to analyse temporal characteristics. The average microvolt of C3 and C4 are drawn on left movements and right movements, respectively. The difference of “C3–C4” in left movements and “C4–C3” in right movements are also drawn for temporal characteristics.

The average microvolt of C3 and C4 are drawn on left movements and right movements, respectively. The difference of “C3-C4” in left movements and “C4-C3” in right movements are drawn for temporal characteristics in Figure 5. Two key sites, C3 and C4 from the sensorimotor region, were chosen to analyse temporal characteristics.

Three key findings from the results in Figure 5 are as follows:

1. Look from the overall, the average microvolt variations of C4 sites are higher than C3 sites on left movements, while the average microvolt variations of C3 sites are higher than C4 sites on right movements. These findings demonstrate the brain's hemisphere lateralization effect during AO.
2. Look from the difference average microvolt variations of (C3-C4) in left movements and (C4-C3) in right movements, the differences of AO-0.25 Hz and AO-1.5 Hz are significantly higher than AO-2.75 Hz and AO-4 Hz for both left movements and right movements. These results confirm that variations during AO captured a pattern of increases with slow-speed movements and decreases with fast-speed movements.
3. In fact, we have calculated the average of difference variations from 1,000 samples for both four different speed modes on left movements and right movements. The results are 0.958 (AO-0.25 Hz), 0.974 (AO-1.5 Hz), 0.048 (AO-2.75 Hz), and 0.112 (AO-4 Hz) on left movements, and 0.907 (AO-0.25 Hz), 0.988 (AO-1.5 Hz), 0.821 (AO-2.75 Hz), and 0.5865 (AO-4 Hz) on right movements. These results suggest some relationships between speed and stimulus direction.

#### 3.3.2. Spectral characteristics

**Figure 6:**
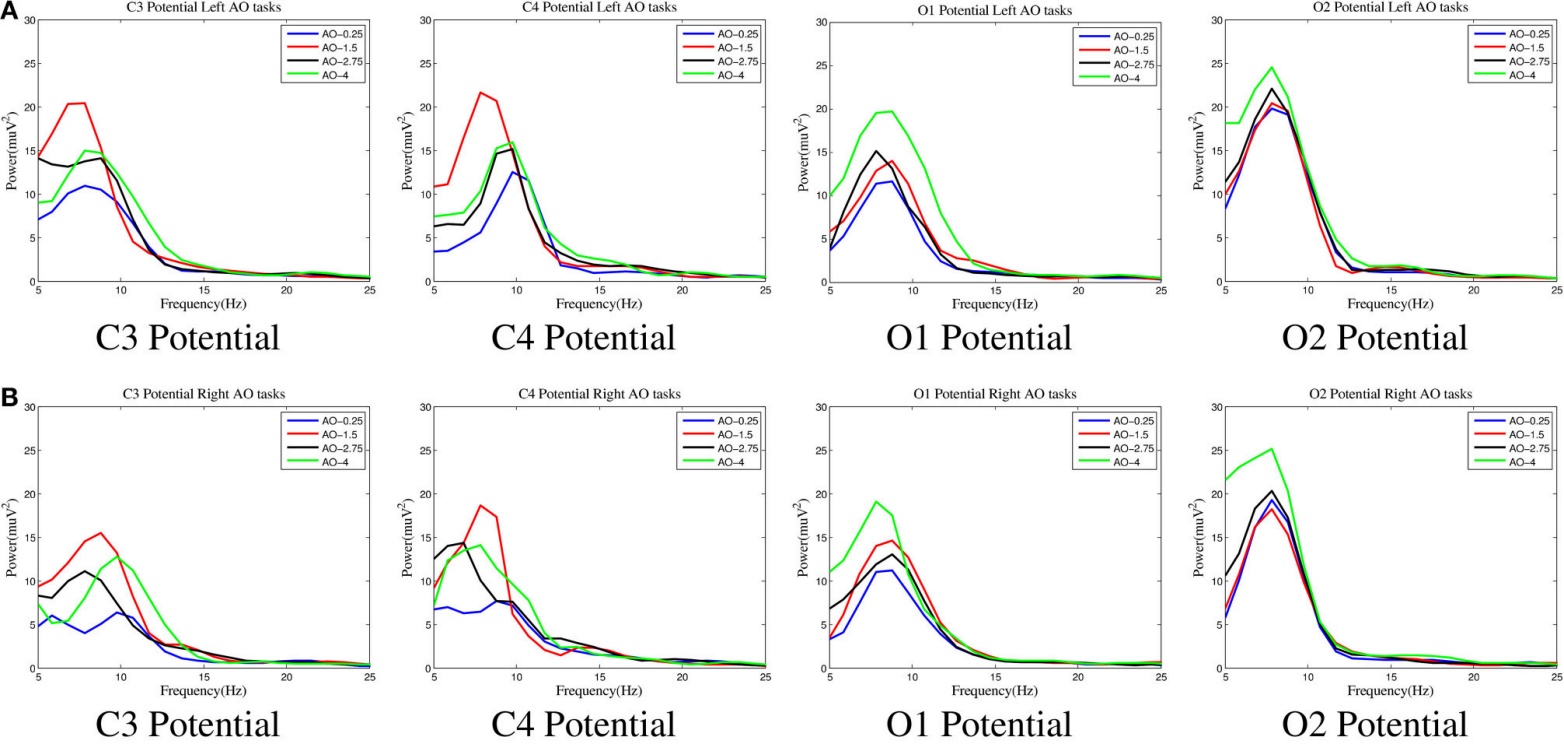
The spectral characteristics of AO in four different speed modes. Four key potentials, C3 and C4 for the sensorimotor region and O1 and O2 for the occipital region, were chosen to analyse the spectral characteristics. The curves for all four AO modes (left movements and right movements) are drawn by “MATLAB R2012a.” **(A)** Left movements of AO. **(B)** Right movements of AO.

Figure 6 shows the spectral characteristics of AO in four different speed modes. Four key potentials, C3 and C4 for the sensorimotor region and O1 and O2 for the occipital region, were chosen to analyse the spectral characteristics.

Three important findings from Figure 6 are as follows:

1. In all of the AO modes, the power varied significantly in the mu range (8–12 Hz) at all C3/C4/O1/O2 potentials, but the variation in the beta range (18–25 Hz) was lower. These results show that the mu range is more sensitive in hMNS during AO.
2. Regardless of whether left or right movements were examined, the power captured variation in the sensorimotor region (C3/C4) with a pattern where the largest was in AO-1.5 Hz, moderate was in AO-2.75 Hz and AO-4 Hz, and the smallest as in AO-0.25 Hz. However, the power captured variation in the occipital region (O1/O2) had an entirely different pattern in which the largest was in AO-4 Hz and the smallest was AO-1.5 Hz, AO-2.75 Hz, and AO-0.25 Hz with no obvious differences. These results suggest that the sensorimotor region was significantly activated at AO-1.5 Hz, whereas the occipital region was significantly activated at AO-4 Hz.
3. Regardless of whether left or right movements were examined, the hemisphere effect of the power difference was prominent in the occipital region (O1/O2) but was not obvious in the sensorimotor region (C3/C4). These results demonstrate that the occipital region is more activated than the sensorimotor region.

#### 3.3.3. Spatio-spectral characteristics

**Figure 7:**
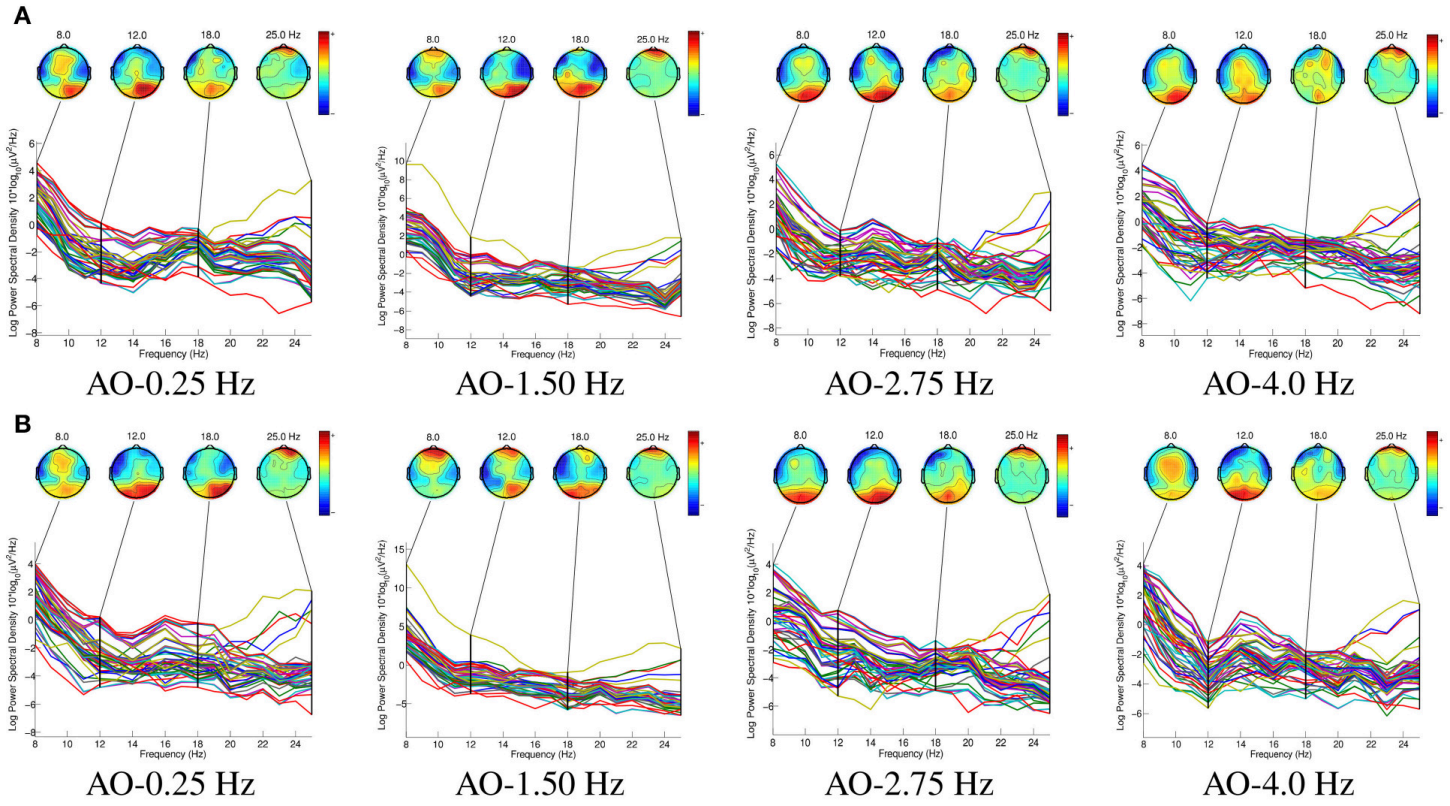
The spatio-spectral characteristics of AO in four different speed modes. The key rhythms, 8, 12, 18, and 25 Hz, were chosen to extract spatio-spectral characteristics from 64 potentials, since the ERD suppressions were induced in the mu (8–12 Hz) and beta (18–25 Hz) ranges. The “BEAM” for all four AO modes(left and right movements) are drawn by “eeglab 14.1.1.” **(A)** Left movements of AO. **(B)** Right movements of AO.

Figure 7 shows the spatio-spectral characteristics of AO in four different speed modes. The key rhythms, 8, 12, 18, and 25 Hz, were chosen to extract spatio-spectral characteristics from 64 potentials, since the ERD suppressions were induced in the mu (8–12 Hz) and beta (18–25 Hz) ranges.

From the results in Figure 7, we find that:

1. In all eight AO modes, the BEAMs showed that the power in the mu range (8–12 Hz) was higher than that in the beta range (18–25 Hz) in the occipital region. These results demonstrate that the mu range plays a predominant role in hMNS activations.
2. In contrast to the ERD suppressions during the four different AO speed modes, the ERD suppressions were stronger at AO-0.25 Hz and AO-1.5 Hz than at AO-2.75 Hz and AO-4 Hz in the sensorimotor region. These findings demonstrate that in the sensorimotor region, slow-speed movements cause greater hMNS activation than fast-speed movements during AO.
3. Comparing left and right movements in one AO mode, the left movements produced more obvious ERD suppressions in the right hemisphere of the brain, whereas the right movements produced more obvious ERD suppressions in the left hemisphere of the brain. These results are due to the mirror effect from the hMNS. However, the movements that subjects observed were also mirror movements. Therefore, the ERD suppressions occurred in the ipsilateral region of the subject's brain relative to the movements of the robot.

## 4. Discussion

This study further explored the brain regions affected during AO, the patterns of relationships between AO speed modes and hMNS activations, and the comparison of AO and MI for constructing BMBIs. AO experiments with different speed modes (experiment 1) and an MI mode (experiment) were explored at the central nervous system level since hMNS activation caused by AO are reflected by ERD suppressions, which are measured via EEG recording. BCI analyses and spatio-spectral-temporal characteristics were used to explore the variations in ERD suppressions. Based on our results, we were able to draw three major conclusions, which are discussed below:

1. The occipital region exhibits higher hMNS activation than the sensorimotor region during AO, but the sensorimotor region shows more robust hMNS activations than the occipital region.
2. hMNS activations initially rise and then fall to a stable level during incremental AO speed modes. A moderate-speed AO produced the best hMNS activations in our results.
3. The BCI accuracy and ITR of moderate-speed AO-BCI produced the same level of MI-BCI as training experience, suggesting a new method for constructing non-invasive BMBIs in healthy individuals.

### 4.1. The affected regions of the brain during AO

According to a statistical study (Molenberghs et al., 2012), the support of mirror neurons, the connections across regions, and the strong mirror properties in the hMNS produce mirror properties in the sensorimotor and occipital regions during AO. In fact, the sensorimotor region is related to action imitation and action presentation. Processing of the subjects' action preparation and spatial orientation for a specific action depend on the sensorimotor region. The occipital region is part of the lateral occipitotemporal cortex (LOTC) (Barton et al., 1996; Kable and Chatterjee, 2006). The LOTC is a set of regions that are thought to function in integrating information and determining the purpose of an action. Several previous studies have found that the LOTC region forms direct and indirect connections with hMNS regions (Weiner and Grill-Spector, 2011; Lingnau and Downing, 2015). Therefore, the occipital, sensorimotor, and “sensorimotor+occipital” regions were adopted for BCI analyses and the extraction of spatio-spectral-temporal characteristic from EEG data during AO in this study.

The accuracies of BCI show that the occipital region exhibits higher ERD suppressions than the sensorimotor region (see Figure 4). Similar results were also found in the BEAMs (see Figure 7). The differences in affected regions during AO found in our study support a previous model of hMNS activation affecting multiple regions (Perry et al., 2010; Frenkel-Toledo et al., 2013). Two main reasons may account for the differences in hMNS activations between the two regions: First, AO paradigms are visually evoked stimuli (Wieser et al., 2016; Park, 2017). Therefore, the visually relevant occipital region first generates power variations (Pegado et al., 2014). After recognition of stimuli, hMNS activations generate power variations in the sensorimotor region (Oberman et al., 2005, 2008). Since the occipital region undergoes rapid and prolonged activation in response to visual stimuli, the degree of activation in the occipital region must be higher than that in the sensorimotor region. Second, the arm movements of the robot turn into attended information during the presentation of AO stimuli (Hwang et al., 2018). Recent studies suggest that attended information increases the activation of mu rhythms (8–12 Hz) in the occipital region during the presentation of visually evoked stimuli (Gray et al., 2015; Sprague et al., 2015). However, distracting information will suppress the activation of the alpha rhythm in the occipital region (Händel et al., 2011; Klimesch, 2012; Zumer et al., 2014). In addition, spectral characteristics and spatio-spatial characteristics suggest that the mu range plays a prominent role in hMNS activations (See Figures 5, 6). Therefore, the attended information provided by the left/right-arm movements of the robot cause more intense power differences in the occipital region than in the sensorimotor region.

Comparing the accuracies of BCI in the three explored regions, the results reflect the robustness of hMNS activation in these regions. The accuracies of BCI in the “sensorimotor+occipital” region remained the same as the sensorimotor region but were significantly different in the occipital region (See Figure 4). Since the activation of the sensorimotor region is caused by ERD suppressions, whereas the activation of the occipital region is caused by both ERD suppressions and attended information, ERD suppressions play a dominant role in the activation of the “sensorimotor+occipital” region. In fact, a large number of sessions must be presented over several days. Therefore, the attended information will change during all sessions. As time passes, the activation of the mu range caused by attended information will gradually reduce due to visual fatigue (Lambooij et al., 2009). However, ERD suppressions remain steady during the presentation of stimuli without influence from visual fatigue (Nam et al., 2011). Therefore, the sensorimotor region is more robust for hMNS activations than the occipital region during AO. The findings in our study of hMNS activations conform to the affected regions and formation principles during AO and support further studies on the hMNS.

### 4.2. The patterns of hMNS activations affected by AO speed modes

We found that the patterns of hMNS activations first rise and then fall to a stable level during incremental-speed modes of AO (See Figure 4). Relationships between speed modes and left/right movements were also found in spatio-spatial characteristics (See Figure 7). The patterns were appropriate for both the sensorimotor region and the occipital region. The reasons why the curves reveal these patterns are as follows. Since AO stimuli in different speed modes were presented in a periodic way in experiment 1, the refractory period of visual stimuli must be considered during the presentation (Huettel and McCarthy, 2000). Previous studies suggest that the inter-stimulus intervals (ISIs) in the refractory period are within 360–2,000 ms for auditory and visual senses (Coch et al., 2005; Brisson and Jolicœur, 2007). Other studies demonstrate that the ISIs of repetitive contents are within 500 ms (Davis et al., 1972; Johannsen and Röder, 2014). In fact, the ISIs in our experiments were 4,000 ms (slow movement), 666.7 ms (moderate movement), 363.6 ms (fast movement), and 250 ms (finalistic movement). Comparing the ISIs of the four speed modes, the fast and finalistic movements exceeded the repetitive content limits of ISI. Therefore, a visual refractory period will appear during the presentation of fast- and finalistic-movement AO tasks. Since the ERD suppressions are also affected by the visual refractory period during AO (Luck et al., 2000; Caravaglios et al., 2015), the hemisphere effects of ERD suppressions will be reduced by the visual refractory period. In other words, increased speed modes of AO within the limits of ISI will promote hMNS activations that benefit from the absence of a visual refractory period.

The patterns suggested that different speed modes of AO define the extent and distribution of hMNS activations. The purpose of our study was to explore the patterns of relationships between AO speed modes and hMNS activations, since the AO speed modes affect action imitation and action presentation during AO. Meanwhile, the speed modes of AO also play important roles in action information integration and the purpose of action in previous studies (Rizzolatti, 2005; Hobson and Bishop, 2016). Therefore, the speed factor must be considered in relevant studies and applications related to action, such as cognitive psychology, rehabilitation treatment, and BCI. The patterns in our study suggest that periodic actions performed at a moderate speed within decent ISI ranges as visual stimuli for observation will contribute to optimized rehabilitation treatment setups for healthy or unhealthy individuals. AO stimuli of moderate speed within decent ISI will also improve the performance of AO-BCI.

Recently, neurological diseases have been remedied by AO strategies, which are good at ameliorating motor recovery (Buccino et al., 2011). For healthy individuals, AO strategies are good at facilitating motor learning and increasing force (Stefan et al., 2005). Actions performed at a suitable speed mode for observation may enhance action motivation and optimize the recruitment of motor function (Porro et al., 2007). Current treatments for chronic stroke and motor impairment patients construct action execution (AE) strategies based on ecological values (Buccino, 2014), which are valid for improving motor function and ameliorating autonomy, such as grasping cups or cleaning the floor (Ertelt et al., 2007). However, since individual patients with neurological diseases usually have clinical impairments, these patients cannot accomplish such AE assignments in daily treatment without help. The limits for individual patients in AE will be remitted by an optimal AO strategy design. Because the AO assignments are visual stimuli, which need patients to focus their attention, AO assignments are easier to accomplish in daily treatment than AE assignments. In addition, regardless of whether AE or AO assignments are used, the treatment strategies of the medical rehabilitative field must be tailored to satisfy the individual needs of each subject. Treatment strategies should be designed with due consideration of environmental, social, and economic factors, as well as the pathology and characteristics of the subject. Our study suggests that an individually tailored speed within a decent ISI range must be considered when constructing rehabilitation AO treatments for individual subjects.

### 4.3. The construction of BMBI based on AO-BCI and MI-BCI

Our study suggests a new way to construct AO-BCI on a humanoid robot platform. Experimental paradigms are defined by parameters such that confounding factors are eliminated by precise settings. Compared with MI-BCI, AO-BCI offers a number of advantages. Typically, MI-BCI is constructed on the self-imaged spontaneous potential of ERD suppressions. Subjects may imagine self-motion, others-motion, wrist movement, arm movement, or hand movement without specific instruction. In addition, the familiarity factor also affects MI-BCI performance, since MI-BCI performance significantly increases for fully fledged subjects (Schroeder and Chestek, 2016; Subramanian et al., 2016). However, AO-BCI, as a visually evoked stimulus, eliminates the uncertainties of spontaneous MI-BCI. Moreover, familiarity in the BCI is controlled by the stimulus design. In experiments 1 and 2, none of the subjects had previous training experience. However, the BCI results of experiment 1 significantly outperformed those of experiment 2 for slow and moderate movements (See Figure 4). A comparison of the results demonstrated that fixed AO modes were less influenced by the subjects' experience, which is another advantage of constructing AO-BCIs.

The performance of AO-BCI and MI-BCI are mismatched in this study since the subjects for MI-BCI lacked MI training. With enough training in MI-BCI, the average accuracy and ITR among subjects can reach 75% and 1.8 bits/min for two classes of MI-BCI (Park et al., 2013). Due to confounding factors, achieving the same level of average accuracy and ITR in AO-BCI has thus far proven difficult. However, by adopting fine-tuned stimuli in AO, as in the present study, the average accuracy and ITR of AO-BCI reached 70% and 1.65 bits/min, which are similar to those of a well-trained MI-BCI (See Figure 4). These findings prompted us to construct a BMBI in healthy subjects using the stimulus paradigms of AO and well-trained MI. In recent studies, the majority of BMBIs were based on invasive signal processing technology and used animal carriers (Tessadori et al., 2012). However, BMBIs are rarely based on healthy human brains given the requirement for non-invasive signal processing techniques. As a system of bi-directional transmission, the BMBI must be built on two kinds of stimulators. The accuracy of performance and the ITR of efficiency must achieve the same level for both transmissions. Designing BMBIs that are influenced by variables remains a meaningful but difficult problem. The construction of BMBIs based on AO-BCI and MI-BCI in our study was based on EEG recordings, which will satisfy the requirement for a non-invasive method in healthy individual subjects.

## 5. Conclusion

Our experimental results suggest that the occipital region exhibits higher but less robust hMNS activations than the sensorimotor region. In addition, the patterns of hMNS activations first rise and then fall to a stable level during incremental-speed modes of AO. Moreover, the creation of an AO-BCI provides a novel means of building BMBI systems in healthy individuals with well-trained MI-BCI. However, as time passes, the subjects' refractory period, fatigue and pressure for waiting will lead limit the EEG recordings. Thus, our results provide only rough patterns of the relationships between AO speed modes and hMNS activations. Further studies with larger experimental trials are needed to explore the exact patterns. More modes of AO are also needed to explore confounding factors. Only then can accurate patterns of AO be completed to study the human brain.

## Author contributions

CZ, TL, and JL designed the experiments; TL and JL completed the experiments; TL analyzed the EEG data; CZ, FC, and TL wrote the paper.

### Conflict of interest statement

The authors declare that the research was conducted in the absence of any commercial or financial relationships that could be construed as a potential conflict of interest.

## References

[B1] Abbott D. F. (2016). Probing the human brain functional connectome with simultaneous EEG and fMRI. Front. Neurosci. 10:302. 10.3389/fnins.2016.00302 27445156 PMC4922049

[B2] Abdi H. Williams L. J. (2010). Principal component analysis. Wiley Interdisc. Rev. Comput. Stat. 2, 433–459. 10.1002/wics.101

[B3] Agnew Z. K. Wise R. J. Leech R. (2012). Dissociating object directed and non-object directed action in the human mirror system; implications for theories of motor simulation. PLoS ONE 7:e32517. 10.1371/journal.pone.0032517 22505995 PMC3323585

[B4] Ang K. K. Chin Z. Y. Wang C. Guan C. Zhang H. (2012). Filter bank common spatial pattern algorithm on BCI competition IV datasets 2a and 2b. Front. Neurosci. 6:39. 10.3389/fnins.2012.00039 22479236 PMC3314883

[B5] Barton J. J. Simpson T. Kiriakopoulos E. Stewart C. Crawley A. Guthrie B.. (1996). Functional MRI of lateral occipitotemporal cortex during pursuit and motion perception. Ann. Neurol. 40, 387–398. 8797528 10.1002/ana.410400308

[B6] Bauer R. Gharabaghi A. (2015). Reinforcement learning for adaptive threshold control of restorative brain-computer interfaces: a Bayesian simulation. Front. Neurosci. 9:36. 10.3389/fnins.2015.00036 25729347 PMC4325901

[B7] Bello J. P. Modroño C. Marcano F. González-Mora J. (2014). The mirror neuron system and motor dexterity: what happens? Neuroscience 275, 285–295. 10.1016/j.neuroscience.2014.06.010 24952330

[B8] Biagi L. Cioni G. Fogassi L. Guzzetta A. Tosetti M. (2010). Anterior intraparietal cortex codes complexity of observed hand movements. Brain Res. Bull. 81, 434–440. 10.1016/j.brainresbull.2009.12.002 20006682

[B9] Blakemore S.-J. Decety J. (2001). From the perception of action to the understanding of intention. Nat. Rev. Neurosci. 2, 561–567. 10.1038/35086023 11483999

[B10] Brisson B. Jolicœur P. (2007). A psychological refractory period in access to visual short-term memory and the deployment of visual–spatial attention: multitasking processing deficits revealed by event-related potentials. Psychophysiology 44, 323–333. 10.1111/j.1469-8986.2007.00503.x 17343714

[B11] Buccino G. (2014). Action observation treatment: a novel tool in neurorehabilitation. Philos. Trans. R. Soc. Lond. B Biol. Sci. 369:20130185. 10.1098/rstb.2013.0185 24778380 PMC4006186

[B12] Buccino G. Gatti R. Giusti M. C. Negrotti A. Rossi A. Calzetti S.. (2011). Action observation treatment improves autonomy in daily activities in Parkinson's disease patients: results from a pilot study. Mov. Disord. 26, 1963–1964. 10.1002/mds.23745 21547952

[B13] Calvo-Merino B. Glaser D. E. Grèzes J. Passingham R. E. Haggard P. (2004). Action observation and acquired motor skills: an fMRI study with expert dancers. Cereb. Cortex 15, 1243–1249. 10.1093/cercor/bhi007 15616133

[B14] Calvo-Merino B. Grèzes J. Glaser D. E. Passingham R. E. Haggard P. (2006). Seeing or doing? Influence of visual and motor familiarity in action observation. Curr. Biol. 16, 1905–1910. 10.1016/j.cub.2006.07.065 17027486

[B15] Caravaglios G. Muscoso E. G. Di Maria G. Costanzo E. (2015). Patients with mild cognitive impairment have an abnormal upper-alpha event-related desynchronization/synchronization (ERD/ERS) during a task of temporal attention. J. Neural Transm. 122, 441–453. 10.1007/s00702-014-1262-7 24947877

[B16] Caspers S. Zilles K. Laird A. R. Eickhoff S. B. (2010). ALE meta-analysis of action observation and imitation in the human brain. Neuroimage 50, 1148–1167. 10.1016/j.neuroimage.2009.12.112 20056149 PMC4981639

[B17] Coch D. Skendzel W. Neville H. J. (2005). Auditory and visual refractory period effects in children and adults: an ERP study. Clin. Neurophysiol. 116, 2184–2203. 10.1016/j.clinph.2005.06.005 16043399

[B18] Davis H. Osterhammel P. Wier C. Gjerdingen D. (1972). Slow vertex potentials: interactions among auditory, tactile, electric and visual stimuli. Electroencephalogr. Clin. Neurophysiol. 33, 537–545. 4117331 10.1016/0013-4694(72)90244-1

[B19] Delorme A. Makeig S. (2004). EEGLAB: an open source toolbox for analysis of single-trial EEG dynamics including independent component analysis. J. Neurosci. Methods 134, 9–21. 10.1016/j.jneumeth.2003.10.009 15102499

[B20] Demandt E. Mehring C. Vogt K. Schulze-Bonhage A. Aertsen A. Ball T. (2012). Reaching movement onset-and end-related characteristics of EEG spectral power modulations. Front. Neurosci. 6:65. 10.3389/fnins.2012.00065 22586364 PMC3345572

[B21] Duffy F. H. Burchfiel J. L. Lombroso C. T. (1979). Brain electrical activity mapping (beam): a method for extending the clinical utility of EEG and evoked potential data. Ann. Neurol. 5, 309–321. 443765 10.1002/ana.410050402

[B22] Ertelt D. Small S. Solodkin A. Dettmers C. McNamara A. Binkofski F.. (2007). Action observation has a positive impact on rehabilitation of motor deficits after stroke. Neuroimage 36, T164–T173. 10.1016/j.neuroimage.2007.03.043 17499164

[B23] Filimon F. Nelson J. D. Hagler D. J. Sereno M. I. (2007). Human cortical representations for reaching: mirror neurons for execution, observation, and imagery. Neuroimage 37, 1315–1328. 10.1016/j.neuroimage.2007.06.008 17689268 PMC2045689

[B24] Franceschini M. Ceravolo M. G. Agosti M. Cavallini P. Bonassi S. Dall'Armi V.. (2012). Clinical relevance of action observation in upper-limb stroke rehabilitation: a possible role in recovery of functional dexterity. a randomized clinical trial. Neurorehabil. Neural Repair 26, 456–462. 10.1177/1545968311427406 22235059

[B25] Frenkel-Toledo S. Bentin S. Perry A. Liebermann D. G. Soroker N. (2013). Dynamics of the EEG power in the frequency and spatial domains during observation and execution of manual movements. Brain Res. 1509, 43–57. 10.1016/j.brainres.2013.03.004 23500633

[B26] Frenkel-Toledo S. Bentin S. Perry A. Liebermann D. G. Soroker N. (2014). Mirror-neuron system recruitment by action observation: effects of focal brain damage on mu suppression. Neuroimage 87, 127–137. 10.1016/j.neuroimage.2013.10.019 24140938

[B27] Gatti R. Rocca M. A. Fumagalli S. Cattrysse E. Kerckhofs E. Falini A.. (2017). The effect of action observation/execution on mirror neuron system recruitment: an fMRI study in healthy individuals. Brain Imaging Behav. 11, 565–576. 10.1007/s11682-016-9536-3 27011016

[B28] Geers A. (1994). Techniques for assessing auditory speech perception and lipreading enhancement in young deaf children. Volta Rev. 96, 85–96.

[B29] Gray M. J. Frey H.-P. Wilson T. J. Foxe J. J. (2015). Oscillatory recruitment of bilateral visual cortex during spatial attention to competing rhythmic inputs. J. Neurosci. 35, 5489–5503. 10.1523/JNEUROSCI.2891-14.2015 25855167 PMC4388917

[B30] Händel B. F. Haarmeier T. Jensen O. (2011). Alpha oscillations correlate with the successful inhibition of unattended stimuli. J. Cogn. Neurosci. 23, 2494–2502. 10.1162/jocn.2010.21557 20681750

[B31] Hobson H. M. Bishop D. V. (2016). Mu suppression–a good measure of the human mirror neuron system? Cortex 82, 290–310. 10.1016/j.cortex.2016.03.019 27180217 PMC4981432

[B32] Huettel S. A. McCarthy G. (2000). Evidence for a refractory period in the hemodynamic response to visual stimuli as measured by MRI. Neuroimage 11, 547–553. 10.1006/nimg.2000.0553 10806040

[B33] Hwang K. Shine J. M. D'Esposito M. (2018). Frontoparietal activity interacts with task-evoked changes in functional connectivity. Cereb. Cortex. 10.1093/cercor/bhy011. [Epub ahead of print]. 29415156 PMC7199886

[B34] Iacoboni M. Molnar-Szakacs I. Gallese V. Buccino G. Mazziotta J. C. Rizzolatti G. (2005). Grasping the intentions of others with one's own mirror neuron system. PLoS Biol. 3:e79. 10.1371/journal.pbio.0030079 15736981 PMC1044835

[B35] Johannsen J. Röder B. (2014). Uni-and crossmodal refractory period effects of event-related potentials provide insights into the development of multisensory processing. Front. Hum. Neurosci. 8:552. 10.3389/fnhum.2014.00552 25120454 PMC4112812

[B36] Kable J. W. Chatterjee A. (2006). Specificity of action representations in the lateral occipitotemporal cortex. J. Cogn. Neurosci. 18, 1498–1517. 10.1162/jocn.2006.18.9.1498 16989551

[B37] Klimesch W. (2012). α-band oscillations, attention, and controlled access to stored information. Trends Cogn. Sci. 16, 606–617. 10.1016/j.tics.2012.10.007 23141428 PMC3507158

[B38] Lambooij M. Fortuin M. Heynderickx I. IJsselsteijn W. (2009). Visual discomfort and visual fatigue of stereoscopic displays: a review. J. Imaging Sci. Technol. 53, 1–14. 10.2352/J.ImagingSci.Technol.2009.53.3.030201

[B39] Lei X. Wu T. Valdes-Sosa P. A. (2015). Incorporating priors for EEG source imaging and connectivity analysis. Front. Neurosci. 9:284. 10.3389/fnins.2015.00284 26347599 PMC4539512

[B40] Lingnau A. Downing P. E. (2015). The lateral occipitotemporal cortex in action. Trends Cogn. Sci. 19, 268–277. 10.1016/j.tics.2015.03.006 25843544

[B41] Luck S. J. Woodman G. F. Vogel E. K. (2000). Event-related potential studies of attention. Trends Cogn. Sci. 4, 432–440. 10.1016/S1364-6613(00)01545-X 11058821

[B42] Maranesi M. Ugolotti Serventi F. Bruni S. Bimbi M. Fogassi L. Bonini L. (2013). Monkey gaze behaviour during action observation and its relationship to mirror neuron activity. Eur. J. Neurosci. 38, 3721–3730. 10.1111/ejn.12376 24118599

[B43] Molenberghs P. Cunnington R. Mattingley J. B. (2012). Brain regions with mirror properties: a meta-analysis of 125 human fmri studies. Neurosci. Biobehav. Rev. 36, 341–349. 10.1016/j.neubiorev.2011.07.004 21782846

[B44] Mukamel R. Ekstrom A. D. Kaplan J. Iacoboni M. Fried I. (2010). Single-neuron responses in humans during execution and observation of actions. Curr. Biol. 20, 750–756. 10.1016/j.cub.2010.02.045 20381353 PMC2904852

[B45] Nam C. S. Jeon Y. Kim Y.-J. Lee I. Park K. (2011). Movement imagery-related lateralization of event-related (de) synchronization (ERD/ERS): Motor-imagery duration effects. Clin. Neurophysiol. 122, 567–577. 10.1016/j.clinph.2010.08.002 20800538

[B46] Neuper C. Scherer R. Wriessnegger S. Pfurtscheller G. (2009). Motor imagery and action observation: modulation of sensorimotor brain rhythms during mental control of a brain–computer interface. Clin. Neurophysiol. 120, 239–247. 10.1016/j.clinph.2008.11.015 19121977

[B47] Newman-Norlund R. D. van Schie H. T. van Zuijlen A. M. Bekkering H. (2007). The mirror neuron system is more active during complementary compared with imitative action. Nat. Neurosci. 10:nn1911. 10.1038/nn1911 17529986

[B48] Oberman L. M. Hubbard E. M. McCleery J. P. Altschuler E. L. Ramachandran V. S. Pineda J. A. (2005). EEG evidence for mirror neuron dysfunction in autism spectrum disorders. Brain Res. Cogn. Brain Res. 24, 190–198. 10.1016/j.cogbrainres.2005.01.014 15993757

[B49] Oberman L. M. Pineda J. A. Ramachandran V. S. (2007). The human mirror neuron system: a link between action observation and social skills. Soc. Cogn. Affect. Neurosci. 2, 62–66. 10.1093/scan/nsl022 18985120 PMC2555434

[B50] Oberman L. M. Ramachandran V. S. Pineda J. A. (2008). Modulation of mu suppression in children with autism spectrum disorders in response to familiar or unfamiliar stimuli: the mirror neuron hypothesis. Neuropsychologia 46, 1558–1565. 10.1016/j.neuropsychologia.2008.01.010 18304590

[B51] Onishi A. Takano K. Kawase T. Ora H. Kansaku K. (2017). Affective stimuli for an auditory p300 brain-computer interface. Front. Neurosci. 11:522. 10.3389/fnins.2017.00522 28983235 PMC5613193

[B52] Orgs G. Dombrowski J.-H. Heil M. Jansen-Osmann P. (2008). Expertise in dance modulates alpha/beta event-related desynchronization during action observation. Eur. J. Neurosci. 27, 3380–3384. 10.1111/j.1460-9568.2008.06271 18598273

[B53] Park C. Looney D. ur Rehman N. Ahrabian A. Mandic D. P. (2013). Classification of motor imagery BCI using multivariate empirical mode decomposition. IEEE Trans. Neural Syst. Rehabil. Eng. 21, 10–22. 10.1109/TNSRE.2012.2229296 23204288

[B54] Park J. (2017). A neural basis for the visual sense of number and its development: a steady-state visual evoked potential study in children and adults. Dev. Cogn. Neurosci. [Epub ahead of print]. 10.1016/j.dcn.2017.02.011 28342780 PMC6969086

[B55] Pegado F. Comerlato E. Ventura F. Jobert A. Nakamura K. Buiatti M.. (2014). Timing the impact of literacy on visual processing. Proc. Natl. Acad. Sci. U.S.A. 111, E5233–E5242. 10.1073/pnas.1417347111 25422460 PMC4267394

[B56] Perry A. Bentin S. Shalev I. Israel S. Uzefovsky F. Bar-On D.. (2010). Intranasal oxytocin modulates EEG mu/alpha and beta rhythms during perception of biological motion. Psychoneuroendocrinology 35, 1446–1453. 10.1016/j.psyneuen.2010.04.011 20493637

[B57] Pineda J. A. Grichanik M. Williams V. Trieu M. Chang H. Keysers C. (2013). EEG sensorimotor correlates of translating sounds into actions. Front. Neurosci. 7:203. 10.3389/fnins.2013.00203 24376395 PMC3858667

[B58] Porro C. A. Facchin P. Fusi S. Dri G. Fadiga L. (2007). Enhancement of force after action observation: behavioural and neurophysiological studies. Neuropsychologia 45, 3114–3121. 10.1016/j.neuropsychologia.2007.06.016 17681358

[B59] Pot E. Monceaux J. Gelin R. Maisonnier B. (2009). Choregraphe: a graphical tool for humanoid robot programming, in The 18th IEEE International Symposium on Robot and Human Interactive Communication (Toyama), 46–51.

[B60] Press C. (2011). Action observation and robotic agents: learning and anthropomorphism. Neurosci. Biobehav. Rev. 35, 1410–1418. 10.1016/j.neubiorev.2011.03.004 21396398

[B61] Rizzolatti G. (2005). The mirror neuron system and its function in humans. Anat. Embryol. 210, 419–421. 10.1007/s00429-005-0039-z 16222545

[B62] Rizzolatti G. Sinigaglia C. (2010). The functional role of the parieto-frontal mirror circuit: interpretations and misinterpretations. Nat. Rev. Neurosci. 11, 264–274. 10.1038/nrn2805 20216547

[B63] Rozzi S. Fogassi L. (2017). Neural coding for action execution and action observation in the prefrontal cortex and its role in the organization of socially driven behavior. Front. Neurosci. 11:492. 10.3389/fnins.2017.00492 28936159 PMC5594103

[B64] Sardouie S. H. Shamsollahi M. B. (2012). Selection of efficient features for discrimination of hand movements from meg using a BCI competition IV data set. Front. Neurosci. 6:42 10.3389/fnins.2012.00042 22485087 PMC3317063

[B65] Schroeder K. E. Chestek C. A. (2016). Intracortical brain-machine interfaces advance sensorimotor neuroscience. Front. Neurosci. 10:291. 10.3389/fnins.2016.00291 27445663 PMC4923184

[B66] Schulte-Rüther M. Markowitsch H. J. Fink G. R. Piefke M. (2007). Mirror neuron and theory of mind mechanisms involved in face-to-face interactions: a functional magnetic resonance imaging approach to empathy. J. Cogn. Neurosci. 19, 1354–1372. 10.1162/jocn.2007.19.8.1354 17651008

[B67] Silsbee P. L. Bovik A. C. (1996). Computer lipreading for improved accuracy in automatic speech recognition. IEEE Trans. Speech Audio Process. 4, 337–351.

[B68] Smith E. G. Bennetto L. (2007). Audiovisual speech integration and lipreading in autism. J. Child Psychol. Psychiatry 48, 813–821. 10.1111/j.1469-7610.2007.01766.x 17683453

[B69] Sprague T. C. Saproo S. Serences J. T. (2015). Visual attention mitigates information loss in small-and large-scale neural codes. Trends Cogn. Sci. 19, 215–226. 10.1016/j.tics.2015.02.005 25769502 PMC4532299

[B70] Stefan K. Cohen L. G. Duque J. Mazzocchio R. Celnik P. Sawaki L.. (2005). Formation of a motor memory by action observation. J. Neurosci. 25, 9339–9346. 10.1523/JNEUROSCI.2282-05.2005 16221842 PMC6725701

[B71] Subramanian L. Morris M. B. Brosnan M. Turner D. L. Morris H. R. Linden D. E. (2016). Functional magnetic resonance imaging neurofeedback-guided motor imagery training and motor training for Parkinson's disease: randomized trial. Front. Behav. Neurosci. 10:111. 10.3389/fnbeh.2016.00111 27375451 PMC4896907

[B72] Summerfield Q. (1992). Lipreading and audio-visual speech perception. Philos. Trans. R. Soc. Lond. B Biol. Sci. 335, 71–78. 1348140 10.1098/rstb.1992.0009

[B73] Tangermann M. Müller K.-R. Aertsen A. Birbaumer N. Braun C. Brunner C.. (2012). Review of the BCI competition IV. Front. Neurosci. 6:55. 10.3389/fnins.2012.00055 22811657 PMC3396284

[B74] Tanji K. Sakurada K. Funiu H. Matsuda K. Kayama T. Ito S.. (2015). Functional significance of the electrocorticographic auditory responses in the premotor cortex. Front. Neurosci. 9:78. 10.3389/fnins.2015.00078 25852457 PMC4360713

[B75] Tessadori J. Bisio M. Martinoia S. Chiappalone M. (2012). Modular neuronal assemblies embodied in a closed-loop environment: toward future integration of brains and machines. Front. Neural Circ. 6:99. 10.3389/fncir.2012.00099 23248586 PMC3520178

[B76] Vogt S. Di Rienzo F. Collet C. Collins A. Guillot A. (2013). Multiple roles of motor imagery during action observation. Front. Hum. Neurosci. 7:807. 10.3389/fnhum.2013.00807 24324428 PMC3839009

[B77] Wang D. Miao D. Blohm G. (2012). Multi-class motor imagery EEG decoding for brain-computer interfaces. Front. Neurosci. 6:151. 10.3389/fnins.2012.00151 23087607 PMC3466781

[B78] Weiner K. S. Grill-Spector K. (2011). Not one extrastriate body area: using anatomical landmarks, hMT+, and visual field maps to parcellate limb-selective activations in human lateral occipitotemporal cortex. Neuroimage 56, 2183–2199. 10.1016/j.neuroimage.2011.03.041 21439386 PMC3138128

[B79] Wieser M. J. Reicherts P. Juravle G. von Leupoldt A. (2016). Attention mechanisms during predictable and unpredictable threat-a steady-state visual evoked potential approach. Neuroimage 139, 167–175. 10.1016/j.neuroimage.2016.06.026 27318217

[B80] Zumer J. M. Scheeringa R. Schoffelen J.-M. Norris D. G. Jensen O. (2014). Occipital alpha activity during stimulus processing gates the information flow to object-selective cortex. PLoS Biol. 12:e1001965. 10.1371/journal.pbio.1001965 25333286 PMC4205112

